# The Eating Disorders Genetics Initiative (EDGI): study protocol

**DOI:** 10.1186/s12888-021-03212-3

**Published:** 2021-05-04

**Authors:** Cynthia M. Bulik, Laura M. Thornton, Richard Parker, Hannah Kennedy, Jessica H. Baker, Casey MacDermod, Jerry Guintivano, Lana Cleland, Allison L. Miller, Lauren Harper, Janne T. Larsen, Zeynep Yilmaz, Jakob Grove, Patrick F. Sullivan, Liselotte V. Petersen, Jennifer Jordan, Martin A. Kennedy, Nicholas G. Martin

**Affiliations:** 1 Department of Psychiatry, University of North Carolina at Chapel Hill, CB #7160, 101 Manning Drive, Chapel Hill, NC 27599-7160 USA; 2 Department of Medical Epidemiology and Biostatistics, Karolinska Institutet, PO Box 281, SE-171 77 Stockholm, Sweden; 3 Department of Nutrition, University of North Carolina at Chapel Hill, Chapel Hill, NC 27599 USA; 4 QIMR Berghofer Medical Research Institute, Locked Bag 2000, Royal Brisbane Hospital, Herston, QLD 4029 Australia; 5 Department of Psychological Medicine, University of Otago, Christchurch, New Zealand; 6 Department of Pathology and Biomedical Science, University of Otago, Christchurch, New Zealand; 7 National Centre for Register-based Research, Aarhus BSS, Aarhus University, Aarhus, Denmark; 8 The Lundbeck Foundation Initiative for Integrative Psychiatric Research, iPSYCH, Aarhus, Denmark; 9 Department of Biomedicine, Aarhus University, Aarhus, Denmark; 10 Center for Genomics and Personalized Medicine, CGPM, and Center for Integrative Sequencing, iSEQ, Aarhus, Denmark; 11 Bioinformatics Research Centre, Aarhus University, Aarhus, Denmark; 12 Department of Genetics, University of North Carolina at Chapel Hill, Chapel Hill, NC 27599 USA

**Keywords:** Anorexia nervosa, Bulimia nervosa, Binge-eating disorder, Eating disorders, Genome-wide association, Psychiatric genetics, Psychiatric genomics consortium, Social media

## Abstract

**Background:**

The Eating Disorders Genetics Initiative (EDGI) is an international investigation exploring the role of genes and environment in anorexia nervosa, bulimia nervosa, and binge-eating disorder.

**Methods:**

A total of 14,500 individuals with eating disorders and 1500 controls will be included from the United States (US), Australia (AU), New Zealand (NZ), and Denmark (DK). In the US, AU, and NZ, participants will complete comprehensive online phenotyping and will submit a saliva sample for genotyping. In DK, individuals with eating disorders will be identified by the National Patient Register, and genotyping will occur using bloodspots archived from birth. A genome-wide association study will be conducted within EDGI and via meta-analysis with other data from the Eating Disorders Working Group of the Psychiatric Genomics Consortium (PGC-ED).

**Discussion:**

EDGI represents the largest genetic study of eating disorders ever to be conducted and is designed to rapidly advance the study of the genetics of the three major eating disorders (anorexia nervosa, bulimia nervosa, and binge-eating disorder). We will explicate the genetic architecture of eating disorders relative to each other and to other psychiatric and metabolic disorders and traits. Our goal is for EDGI to deliver “actionable” findings that can be transformed into clinically meaningful insights.

**Trial registration:**

EDGI is a registered clinical trial: clinicaltrials.gov NCT04378101.

## Background

We describe the *Eating Disorders Genetic Initiative* (*EDGI*) designed to expand genomic discovery across the three major eating disorders (AN [anorexia nervosa], BN [bulimia nervosa], and BED [binge-eating disorder]). EDGI builds on a previous genome-wide association study (GWAS) by the Eating Disorders Working Group of the Psychiatric Genomics Consortium (PGC-ED) as part of the Anorexia Nervosa Genetics Initiative (ANGI) that identified 8 significant loci associated with AN and intriguing genetic correlations (r_g_) with both psychiatric and metabolic traits, strongly suggesting that AN is a metabo-psychiatric disorder [1].

Although foundational, ANGI focused only on AN. Despite decades of work confirming the heritability, morbidity, mortality, and burden associated with BN and BED [2–6], no GWAS of these disorders exist. EDGI is designed to ascertain, phenotype, and genotype large AN, BN, and BED samples along with ancestrally matched controls across the United States (US), Australia (AU), New Zealand (NZ), and Denmark (DK). We will apply advanced analytic strategies to test and refine an etiological model of eating disorders, explicate heterogeneity, and preliminarily explore environmental risk factors for eating disorders. EDGI represents the next logical step in eating disorder genomics.

Our work emerges from a testable conceptualization of eating disorders as arising from a shared vulnerability to a genetically influenced core component (e.g., dysregulated appetite) that is further differentiated across clinical presentations of AN, BN, and/or BED by differing genetic predispositions to dimensional eating disorder behaviors (e.g., binge eating, vomiting, excessive exercise), BMI, personality, comorbid psychopathology, physical activity, and metabolic and anthropometric traits. We propose that this palette of genetic risk is further influenced by environmental factors that affect emergence, course, and outcome of the eating disorder [7]. EDGI will empirically test and refine this model. Importantly, we will achieve greater diversity by ascertaining ancestrally diverse cases reflecting known epidemiological distributions of eating disorders.

## Method

### Specific aims

Aim 1. Recontact. We will recontact ~ 4000 participants from a previous AN genetic study (ANGI) in the US, AU, and NZ to obtain deeper phenotypic information online about course of illness, comorbid psychiatric conditions, treatment response, healthcare utilization, and quality of life. *Deliverables: Rich phenotypic information to expand clinical description of former ANGI participants. Biological samples will not be collected from prior ANGI participants as they were previously genotyped.*

Aim 2. Ascertainment. Ascertainment of 14,500 new eating disorder cases and 1500 controls. Using economical and online recruitment strategies in the US, AU, and NZ, we will ascertain, phenotype, and genotype ~ 2800 new AN cases; ~ 3250 BN cases; ~ 4050 BED cases and 900 controls. In DK, a total of 4400 cases and 600 controls will be identified from the Danish National Patient Register and genotyping will occur with samples from archived bloodspots from birth. Combined with existing PGC-ED samples (total 35,000 cases, 100,000 controls), this will define our GWAS analysis set. *Deliverables: Analysis of deep phenotypic information from individuals with AN, BN, and BED plus analysis-ready datasets for genetic analyses in Aims 3 and 4.*

Aim 3. Within disorder analyses. We will conduct pre-planned GWAS for AN, BN, BED, any eating disorder, and component behaviors plus a specific set of post-GWAS analyses. *Deliverables: (a) GWAS meta-analysis of EDGI with existing PGC-ED AN, BN, and BED data; b) combined eating disorder GWAS and component behavior GWAS; (c) comprehensive suite of post-GWAS analyses; (d) data deposit per current legal and regulatory standards.*

Aim 4. Cross disorder analyses. Following Aims 1–3, we will test/refine our conceptualization of eating disorders, particularly their inter-relations and connections with other traits. (a) We will calculate r_g_ among AN, BN, BED, and an array of psychiatric, metabolic, anthropometric, physical activity, and educational phenotypes; (b) use Mendelian randomization to investigate putative causal relations; (c) use multi-trait conditional and joint analysis to determine genetic associations that are not driven by confounding correlated traits; (d) identify SNP-associations that are specific to AN, BN, or BED (i.e., that deviate from genome-wide pleiotropy); (e) generate multi-trait genetic risk scores (GRS) to improve out-of-sample prediction; and (f) link DK genotypes with population registers to preliminarily explore genotype (GRS) × environment interactions. *Deliverables: (a) Empirical answers to core research questions about eating disorder etiology including detailed clarification of the direct genetic and correlated forces that drive the observed relationships among eating disorders and between eating disorders and other traits; (b) insights into how genes and environment interact to influence eating disorder risk.*

### Participants

For Aim 1, we will recontact ~ 4000 ANGI participants from the US, AU, and NZ to obtain deeper online phenotyping on onset, course of illness, comorbid psychiatric conditions, treatment response, healthcare utilization, and health-related quality of life. For Aim 2, we will ascertain, phenotype, and genotype ~ 4500 individuals with AN, ~ 5950 individuals with BN, ~ 4050 individuals with BED, and 1500 controls.

### Inclusion criteria

US, AU, NZ case definition*.* Inclusion criteria for AN, BN, and BED cases (female and male) are based on DSM-5 [8] criteria as determined by the ED100K.V3 [9]. Minimum inclusion ages vary by country and range from 13 to 18, with no upper age limit.

DK Case definition*.* Cases are identified who have an ICD [10] diagnostic code of F50.0, F50.1 (AN), F50.2, or F50.3 (BN) in the Danish National Health Register.

US, AU, NZ control definition*.* Controls in US, AU, and NZ are broadly matched to cases by age, ancestry, have lifetime adult minimum BMI > 18.55 kg/m^2^, and no history of an eating disorder or disordered eating behaviors.

DK control definition*.* Controls matched for age and sex in DK with no eating disorder history (F50, F50.1, F50.2, F50.3, F50.8, or F50.9) are identified from population registers.

### Recruitment

US, AU, NZ recruitment. The three countries with active recruitment use a multi-pronged recruitment approach including: outreach to eating disorders clinicians and programs across the countries, traditional media (press releases, television, radio, and newspaper announcements), and social media (websites, Facebook, Twitter, Instagram, LinkedIn, podcasts). Each country also recruited a diverse team of EDGI Ambassadors (individuals with lived experience, parents and relatives, clinicians) who were comfortable telling their story and why they chose to participate in EDGI. Ambassadors were invited who represented a range of eating disorders, body shapes and sizes, genders, and ancestries. These people contributed interviews and commentaries that were used in study launches and advertising, and which formed part of the educational resources provided via EDGI websites in each country.

### Procedure

Procedures differ slightly across sites due to requirements of the local ethical committees. Below we summarize the common procedures first, followed by site-specific variations.

US, AU, NZ procedures. Individuals who are interested in learning more about EDGI or participating, visit the country-specific website (edgi.org, edgi.org.au, edgi.nz). Participants click the “Take Our Survey” link. Slight differences exist in consent procedures due to local ethical requirements. After providing online informed consent, participants complete the ED100K.V3 questionnaire. Surveys are presented in REDCap [11] in the US and NZ and in Qualtrics (https://www.qualtrics.com) in AU. Embedded algorithms determine if they meet inclusion criteria (lifetime DSM-5 criteria for AN, BN, or BED). If they do meet criteria, those in the US are required to provide a second consent for further participation.

Enrolled participants complete a battery of assessments (see Table 1) and are sent a saliva collection kit in the mail. Individuals follow the instructions to submit a saliva sample and return their “spit kit” to the laboratory in the post. Completion of the questionnaires and receipt of the saliva kit in the mail marks completion of the study.

**Table 1:** EDGI Assessment Battery

Assessment	Cases (ED)/ Controls (CO)	Domain
ED100K [9]	ED/CO	Lifetime eating disorder diagnoses
Eating Disorder Examination-Questionnaire [12]	ED only	Current eating disorder symptoms (last 28 days)
ED-Quality of Life [13, 14]	ED only	Eating disorder-specific health related quality of life
Short Form Health Survey-12 [15]	ED/CO	General mental and physical health
Eating disorder treatment history	ED only	Lifetime eating disorder-related healthcare utilization
Obsessive-Compulsive Inventory-R [16]	ED/CO	Obsessive-compulsive symptoms
PHQ-9 [17]	ED/CO	Current depression symptoms
GAD-7 [18]	ED/CO	Current anxiety symptoms
GLAD Depression and Anxiety https://gladstudy.org.uk/	ED/CO	Detailed assessment of major depressive disorder and generalized anxiety disorder
AUDIT [19]	ED/CO	Alcohol use (adapted for lifetime measurement)
Heaviness of Smoking/Vaping [20]	ED/CO	Nicotine use
DUDIT [21]	ED/CO	Drug use (adapted for lifetime measurement)
Life Events Checklist for DSM-5 [22]	ED/CO	Life events and trauma history
Compulsive Exercise Test [23]	ED/CO	Driven exercise
Multidimensional Perfectionism Scale [24]	ED/CO	Perfectionism

Site-specific procedures (US, AU, NZ)*. Age*. The US is enrolling participants aged 15 and older. Parental consent is required for any participant under age 18 (21 in Puerto Rico). Parental consent is required for participants under age 18 in AU. Separate procedures obtain online consent from parents and assent from children. In AU, individuals aged 13 and older can participate. Parental consent is required for any participant under age 18. NZ is enrolling participants aged 16 and older; parental consent is not required. *Gift cards*. In the US and NZ, participants are given gift cards/vouchers once their questionnaires are complete and spit kit confirmed returned to the lab. Australian law prohibits incentives for participating in research.

Denmark procedures. *Study population*: All children born in DK to Danish-born parents from 1981 to 2009 who were alive and living in DK on their 1st birthday (*N* = 1,717,316) constitute the source population for the iPSYCH register sample which includes all the individuals with ICD-10 diagnoses of F50.0 and F50.1 [25]. Furthermore, diagnoses of F50.2 and F50.3 are being identified in the iPSYCH sample. However, it is not possible to ascertain BED in DK as it does not have a unique diagnostic code in ICD-10. Additional control data are available from > 50,000 controls via the iPSYCH collaboration [25].

*Data sources*: Genomic data for all individuals are obtained from genotyping of blood samples from Guthrie cards, collected days after birth and stored at Statens Serum Institut (SSI). DNA will be extracted and whole genome amplified in triplicate [26]. Individuals with an AN diagnosis before 2013 have been genotyped as part of ANGI. EDGI will extend collection to include birth cohorts from 2006 to 2009 and new AN cases from 2014 to 2016. All approvals by ethics committees and Data protection Agencies have already been obtained by the iPSYCH collaboration, and aligned with General Data Protection Regulation (GDPR) requirements.

*Linking to population registers*: DK participants cannot be contacted per Danish law, so we cannot administer the questionnaire battery. However, we can link genomic data to a wealth of Danish national registers. We highlight that domains relevant to this proposal are capturable by registers. We will harmonize to the extent possible between the US, AU, NZ information and that from DK registers. *Inpatient treatment*: admission/discharge dates, medical specialty (i.e., psychiatry), primary diagnosis and ~ 20 secondary diagnoses (ICD codes) [27, 28] since the 1970s. *Captures: course of illness, treatment utilization, somatic and psychiatric comorbidity. Outpatient psychiatric treatment*: date, place, primary/secondary ICD codes since early 2000s. *Captures: treatment utilization and psychiatric comorbidity. Prescription drugs*: all redeemed prescriptions as ATC codes (e.g., fluoxetine is N06AB03) since mid 1990s. *Captures: treatment utilization, diagnosis. Death register*: official causes of death–direct cause, all contributing causes, and relevant comorbidities including suicide (ICD codes) since 1970s. *Captures: mortality. Birth register*: mother’s parity, parental ages, place of birth, delivery data (gestational age, birth weight-length, delivery type, presentation, obstetrical complications, and 1, 5, and 10 min Apgar scores). *Captures: environmental risk factors. Multi-generation register* connects people into pedigrees to create comprehensive family history (parents, siblings, and children of a person also have all of the above data) and estimation of severe stressors (death of a parent) since the 1960s. *Captures: family history. Demography*: sociodemographic characteristics including living situation (alone, cohabiting, marital status), education levels, employment, income, and sick leave/disability (from 1980 onward). *Captures: quality of life (QOL), disease burden.*

DNA extraction and genotyping*.* DNA extraction and GWAS genotyping are standard. US, AU, and NZ samples will be genotyped together. In DK, sample preparation and genotyping will be performed at SSI as described fully elsewhere [29]. We will use the most contemporary chip appropriate for diverse ancestry populations when genotyping occurs.

### Self-report measures

Table 1 presents the assessment instruments and the domains captured. These assessments are administered to all previous ANGI participants (Aim 1) and all individuals recruited in the US, AU, and NZ. In the US, the battery is available in English or Spanish. The battery has been translated into several languages and we are encouraging other sites around the world to adopt the standard battery for future comparability.

ED100K.V3 [9] questionnaire is a self-report, ED assessment based on the Structured Clinical Interview for DSM-5, Eating Disorders. Items assess DSM-5 criteria for AN, BN, BED, and other specified feeding and eating disorders (OSFED). The ED100K-V1 was found to be a valid measure of eating disorders and eating disorder behaviors. Positive predictive values for AN Criterion B, Criterion C, and binge eating ranged from 88 to 100%. Among women who had a negative screen, the probability of not having these criteria or behaviors ranged from 72 to 100%. The correlation between questionnaire and interview for lowest illness-related BMI was 0.91. *Captures: diagnosis, symptoms, course of illness, diagnostic fluctuation.*

Eating Disorders Examination Questionnaire, version 6 (EDE-Q [12]; 28-items). The EDE-Q, which is based on and correlates well with the EDE [30], will assess current eating disorder symptoms. *Captures: current ED pathology.*

Eating Disorder Health-Related Quality of Life (ED-QOL) [13, 14]. The ED-QOL is a health-related quality of life questionnaire designed specifically for eating disorders. The 26-item instrument has excellent test-retest reliability and convergent validity. *Captures: eating disorder-related quality of life.*

Short Form Heath Survey (SF-12) [15] is a standardized widely-used measure of impairment associated with physiological and psychological health conditions. Yields two weighted scales: Physical Component Summary Scale (PCS) and Mental Component Summary Scale (MCS). *Captures: general health-related quality of life.*

Eating Disorders Treatment History. The Eating Disorders Treatment History section of the assessment battery was modeled after the New Zealand COST study (The Costs Of Eating Disorders In NZ online survey) which incorporated elements of both the Australian Butterfly Foundation [31] and beat [32] economic surveys. Specifics of treatment options have been tailored to reflect the healthcare system of each participating country (US, AU, NZ).

Obsessive-Compulsive Inventory-Revised (OCI-R) [16]. The OCI-R assesses obsessions and a variety of compulsions using 6 subscales. Each item is scored on a 5-point scale (0–4 points). The total score (range: 0–72) is the sum of the scores on all items. Scores of 21 and higher might indicate obsessive compulsiveness. *Captures: obsessive-compulsive tendencies.*

The Patient Health Questionnaire 9 (PHQ-9) [17] is a 9-item, self-administered version of the PRIME-MD diagnostic instrument for common mental disorders. The nine items are based on the nine DSM-IV criteria for major depressive disorder and are scored as “0” (not at all) to “3” (nearly every day). The PHQ-9 has been found to be a reliable and valid measure of depression severity.

The Generalized Anxiety Disorder 7 (GAD-7) [18] is a 7-item, self-report questionnaire to screen for generalized anxiety disorder. Each symptom is scored on a 3-point scale: “not at all” (0), “several days” (1), “more than half the days” (2), or “nearly every day” (3). Items are then summed to create a symptom severity score. The GAD-7 is a reliable and valid measure of anxiety.

GLAD Questionnaire (sections B and C) assesses lifetime major depressive disorder and anxiety disorders. This is being used in the UK GLAD study (https://gladstudy.org.uk/), which is ascertaining and genotyping 40 K with major depressive disorder and anxiety disorders. *Captures: lifetime depression and anxiety disorders and symptoms.*

Alcohol Use Disorders Identification Test: Self-Report Version (AUDIT) [19]. Individuals are asked about the frequency of alcohol intake, typical drinking patterns, presence of binge drinking, and the social impact of drinking. We received permission to modify the wording to assess lifetime drinking (when at worst). The assessment is 10 questions, each of which scores between 0 and 4 points. The scores of each question are summed (total possible = 40). Hazardous or harmful use is indicated by scores > 7 in men and > 6 in women. *Captures: problematic alcohol use at the time of heaviest use.*

Heaviness of Smoking Index (HSI) [20]. The HSI uses two questions to assess heaviness of smoking during the period when the individual was smoking the most: number of cigarettes per day and time to the first cigarette of the day*.* Vaping questions were added to capture current trends. *Captures: heaviest smoking and vaping.*

Drug Use Disorders Identification Test (DUDIT) [21]. The DUDIT is a self-report assessment to screen for drug use and dependence. The assessment is 11 questions and the total score is defined as the sum of all question responses. We received permission to modify the wording to assess lifetime drinking(when at worst). *Captures: problematic drug use at the time of heaviest use.*

Life Events Checklist for DSM-5 (LEC-5) [22] is a standard self-report was used to screen for 16 traumatic events that an individual might have experienced during their lifetime. It also includes an item to capture any other event not included in the 16. We added additional questions about emotional and physical neglect/abuse during childhood. Participants were also asked about the ages at which they first and last experienced each event.

Compulsive Exercise Test (CET) [23]. The CET is a 24-item, multidimensional assessment of compulsive exercise. Items are scored from 0 (“never true”) to 5 (“always true”). In addition to a global scale, subscales measure avoidance, weight control, mood improvement, lack of exercise enjoyment, and exercise rigidity. *Captures: driven exercise.*

Multidimensional Perfectionism Scale (MPS) [24]. We used a reduced version of the MPS, consisting of 12 items and three subscales (concern over mistakes, doubts about actions, personal standards). The 12 items were previously selected by Bulik and colleagues [33] out of the full MPS based on research findings and communication with the scale developers.

Saliva sampling. Saliva samples are collected with Isohelix saliva collection kits and returned to labs at UNC (US), QIMR Berghofer (AU), or University of Otago (NZ).

### Planned data analysis

Aims 1 and 2. *Analysis of phenotypic data:* We will analyze the rich phenotypic data from the US, AU, and NZ in collected in ***Aims 1–2*** to compare onset, course of illness, comorbid psychiatric conditions, treatment response, healthcare utilization, and health-related quality of life across AN, BN, and BED cohorts. We will use generalized linear modeling to compare outcomes among AN, BN, and BED. For continuous outcomes with a normal distribution we will use ANOVA methods, for count outcomes we will use Poisson regression, for categorical outcomes we will use multinomial logistic regression, for ordinal outcomes we will use ordinal logistic regression, and where indicated for time-scale outcomes we will use Cox proportional hazards regression. Post hoc analyses corrected for multiple testing will be used to follow-up on significant omnibus results.

Aim 3. We will conduct GWAS for AN, BN, and BED independently, combined in an all-eating disorder analysis, and GWAS of component behaviors and traits. We will also progress through a series of pre-planned post-GWAS analyses, and conduct external replications.

- *GWAS with the PGC Ricopili pipeline.* To develop a robust way to process hundreds of GWAS data sets rapidly and consistently, a PGC team led by Drs S. Ripke and M. Daly developed, the “Ricopili” software system. Ricopili consists of modules for: Pre-imputation (QC), principal components analysis (PCA), Imputation, and Meta-analysis which we use to flexibly develop customized pipelines for genetic analysis, most of which use PLINK2 [34] and other published tools for imputation and genetic analysis [35, 36]. Ricopili has been installed on the DK GenomeDK cluster as well so that genetic QC, imputation, and analysis of all EDGI data will be harmonized.

Ricopili has been used in almost all primary PGC papers. Briefly, genotype calling uses standard software [37]. QC and imputation are performed per dataset so that these procedures are performed on technically homogeneous sets of cases and controls. QC parameters for retaining SNPs and subjects: SNP missingness < 0.05 (before subject removal); subject missingness < 0.02; autosomal heterozygosity deviation (|*F*_*het*_| < 0.2); SNP missingness< 0.02 (after subject removal); difference in SNP missingness between cases and controls < 0.02; and SNP HWE (*P* > 10^− 6^ in controls and *P* > 10^− 10^ in cases). Slight differences in QC parameters are employed in DK. Alternative thresholds can be implemented if needed. Subjects are further screened for fingerprinting mismatch, relatedness to any other subject ($\hat{\pi}$ < 0.2), unusual homozygosity, and sex mismatch. For US, AU, and NZ, imputation will be on TOPMEd [38] (*N* = 65 K 30x WGS) and for DK on HRC. ChrX imputation and analysis is fully implemented (excluding chrX SNPs with missingness ≥0.05 or HWE *P* < 10^− 6^ in females, imputation done separately in males and females). The PGC central analysis team supports other analyses including runs of homozygosity [39–41], segmental sharing of IBD regions [42, 43], GxG interactions, and conditional analyses as complementary ways to identify loci. Results are disseminated via the PGC (http://pgc.unc.edu/downloads).

Ancestry will be assessed using PCA for each subject, mapped relative to reference samples of known ancestry (Illumina GWAS on 1000 Genomes EUR, AFR, EAS, SAS, and AMR samples [44] plus Genome-EUTWIN). Common variants are relatively old and so trait associations are expected largely to be shared across global ancestries. Population-specific LD patterns that result from drift or natural selection mean that cross-ancestry analyses can help fine-map associated loci. Estimated cross-ancestry r_g_ are > 0.75 for other common diseases [45]. The PGC has found that the genetic correlation for schizophrenia in EUR and EAS samples was indistinguishable from 1 (0.98, SE 0.03). Consistent with our intention to include non-EUR ancestries, we will use established PGC cross-ancestry analytical approaches. The exact method depends on the ancestries represented in the final data, and the extent of admixture. We will have direct access to primary genotypes, and so can apply mixed modelling approaches using GRMs as implemented in GCTA and other packages. More trans-ancestry analysis methods are expected to be developed as larger mixed ancestry data sets become available, building upon trans-ancestry meta-analysis [46] trans-ancestry LD score regression, the POPCORN method [47]. The different LD architectures underpin these approaches. If we were to do these analyses now, to account for genetically diverse data with potential hidden structures, we would use a general mixed model (BOLT-LMM) [48], supplemented, if necessary, with the big-K, small-K matrices (that account for closer and more distant genetic relationships) [49].

b) *Disorder-specific GWAS.* We will conduct disorder-specific GWAS for AN, BN, and BED combining EDGI with existing PGC-ED data using imputed variant dosages and an additive model. Covariates nominally associated with the phenotype in univariate analysis (*P* < 0.05) and five ancestry principal components will be included in GWAS. All cohorts will be meta-analyzed with an inverse-variance weighted fixed-effect model. We are likely to filter our GWAS results with minor allele frequency (MAF) ≥ 0.01 and INFO score ≥ 0.70 (indicating “high-quality”). We anticipate aggregate sample sizes for EDGI, PGC-ED, and UKBiobank of: (AN = 22,000, BN = 7000, BED = 6000, and controls = 100,000). To account for genetically diverse data with potential hidden structures and case-control, we will use a general mixed model (BOLT-LMM) [48], and if necessary use big-K/small-K matrices (that account for closer and more distant genetic relationships) [49].
c) *Combined ED GWAS.* We will conduct GWAS meta-analysis of all EDs and of component behaviors, increasing power to identify genetic risk factors that are common across the three disorders.
d) *Post-GWAS analyses.* We will conduct a palette of standard post-GWAS analysis to maximize information yield and interpretability. For examples of standard post-GWAS analytic approaches see Watson et al. [1]

Aim 4. We will calculate r_g_ among AN, BN, BED, and an array of psychiatric, metabolic, anthropometric, physical activity, and educational phenotypes. We will (b) use Mendelian randomization to investigate putative causal relations; (c) use multi-trait conditional and joint analysis to determine genetic associations that are not driven by confounding correlated traits; (d) identify SNP-associations that are specific to AN, BN or BED (i.e., that deviate from genome-wide pleiotropy); (e) generate multi-trait GRS to improve out-of-sample prediction; and (f) link DK genotypes with population registers to preliminarily explore genotype (GRS) × environment interactions.

## Discussion

In addition to the science proposed in EDGI, we will be creating national and international data and sample resources for researchers around the world to use in pursuit of related research questions. EDGI has also been established as an international prototype for other countries to follow. Translation of assessments and cloning of procedures are ensuring that future data and sample collection globally will be readily harmonizable. Although our proposed analytic aims are dense, the data that EDGI yield will be informative for additional research questions such as: 1) genetic factors influencing stability versus fluctuation of eating disorder clinical presentation (e.g., can one predict on the basis of genetics who is at risk for developing BN or BED after experiencing AN); 2) potentially, precision-medicine questions regarding identification of optimal interventions informed by genotype. Currently, only two FDA-approved medications for eating disorders exist—fluoxetine for BN (approved in 2002) and lisdexamfetamine for BED (approved in 2015). No FDA approved medications for AN exist. None of these medications was developed based on knowledge of underlying disease biology. Ultimately we hope that our work will yield information on critical biological pathways that may point toward drug discovery or repurposing that could aid in reversing the tenacity and lethality of these illnesses.

## Data Availability

Our liberal data and analysis sharing principles will make phenotypic and genotype data and scripts widely available for access by other scientists to maximize utility of our investigation. The datasets generated and/or analyzed from the US, AU, and NZ will be available in the National Data Archive (https://nda.nih.gov/). Genomic data access is made possible by the Psychiatric Genomics Consortium Data Access Committee. Step-by-step procedures are thoroughly described here (https://www.med.unc.edu/pgc/shared-methods/open-source-philosophy/). DNA samples from the US, AU, and NZ will be available from the NIMH Repository and Genomics Resource (https://www.nimhgenetics.org/order-biosamples/how-to-order-biosamples). Due to Danish law, data and samples and data cannot be shared with researchers outside of the country.

## Abbreviations

AFR: African
AMR: Ad Mixed American
AN: Anorexia nervosa
ANGI: Anorexia Nervosa Genetics Initiative
ANOVA: Analysis of variance
ATC: Anatomical Therapeutic Chemical Classification System
AU: Australia
AUDIT: The Alcohol Use Disorders Identification Test
BED: Binge-eating disorder
BMI: Body mass index
BN: Bulimia nervosa
CET: Compulsive Exercise Test
COST: The Cost of Eating Disorders in New Zealand study
DK: Denmark
DNA: Deoxyribonucleic acid
DSM-IV: Diagnostic and Statistical Manual of Mental Disorders 4th Edition
DSM-5: Diagnostic and Statistical Manual of Mental Disorders 5th Edition
DUDIT: The Drug Use Disorders Identification Test
EAS: East Asian
ED100K-V1: Eating Disorders 100,000 Questionnaire Version 1
ED100K.V3: Eating Disorders 100,000 Questionnaire Version 3
EDE-Q: Eating Disorders Examination-Questionnaire
EDGI: Eating Disorders Genetics Initiative
ED-QOL: Eating Disorders Quality of Life
EUR: European
FDA: Food and Drug Administration
GAD-7: Generalized Anxiety Disorder 7
GCTA: Genome-wide Complex Trait Analysis
GDPR: General Data Protection Regulation
GLAD: Genetic Links to Anxiety and Depression study
GRM: Genetic relationship matrix
GRS: Genetic risk score
GWAS: Genome-wide association study
HRC: Haplotype Reference Consortium
HSI: Heaviness of Smoking Index
HWE: Hardy-Weinberg equilibrium
IBD: Identical by descent
ICD: International Classification of Diseases
LD: Linkage disequilibrium
LEC-5: Life Events Checklist for DSM-5
LMM: Linear mixed model
MAF: Minor allele frequency
MCS: Mental Component Summary Scale
MPS: Multi-dimensional Perfectionism Scale
NZ: New Zealand
OCI-R: Obsessive Compulsive Inventory-Revised
OSFED: Other Specified Feeding and Eating Disorders
PCA: Principal components analysis
PCS: Physical Component Summary Scale
PGC-ED: Eating Disorders Working Group of the Psychiatric Genomics Consortium
PHQ-9: Patient Health Questonnaire-9
QC: Quality control
QIMR: Berghofer Queensland Institute of Medical Research
QOL: Quality of life
r_g_: Genetic correlation
SAS: South Asian
SF-12: Short Form-12
SNP: Single nucleotide polymorphism
SSI: Statens Serum Institut
TOPMed: Trans-omics for Precision Medicine
UNC: University of North Carolina
US: United States of America
